# A Small World Graph Approach for an Efficient Indoor Positioning System

**DOI:** 10.3390/s21155013

**Published:** 2021-07-23

**Authors:** Max Lima, Leonardo Guimarães, Eulanda Santos, Edleno Moura, Rafael Costa, Marco Levorato, Horácio Oliveira

**Affiliations:** 1 Institute of Computing, Federal University of Amazonas, Manaus 69080-900, Brazil; mw_ac@icomp.ufam.edu.br (M.L.); emsantos@icomp.ufam.edu.br (E.S.); edleno@icomp.ufam.edu.br (E.M.); 2 Institute of Innovation, Research, and Scientific Development of Amazonas, Manaus 69010-001, Brazil; leonardo.guimaraes@ipdec.org; 3 Education Technologies, Positivo Technologies, Curitiba 81350-000, Brazil; rkcosta@positivo.com.br; 4 Computer Science Department, University of California, Irvine, CA 92697, USA; levorato@uci.edu

**Keywords:** indoor positioning systems, k-nearest neighbors, hierarchical small world graphs

## Abstract

The main goal of an Indoor Positioning System (IPS) is to estimate the position of mobile devices in indoor environments. For this purpose, the primary source of information is the signal strength of packets received by a set of routers. The fingerprint technique is one of the most used techniques for IPSs. By using supervised machine learning techniques, it trains a model with the received signal intensity information so it can be used to estimate the positions of the devices later in an online phase. Although the k-Nearest Neighbors (kNN) is one of the most widely used classification methods due to its accuracy, it has no scalability since a sample that needs to be classified must be compared to all other samples in the training database. In this work, we use a novel hierarchical navigable small world graph technique to build a search structure so the location of a sample can be efficiently found, allowing the IPSs to be used in large-scale scenarios or run on devices with limited resources. To carry out our performance evaluation, we proposed a synthetic IPS dataset generator as well as implemented a complete real-world, large-scale IPS testbed. We compared the performance of our graph-based solution with other known kNN variants, such as Kd-Tree and Ball-Tree. Our results clearly show the performance gains of the proposed solution at 98% when compared to the classic kNN and at least 80% when compared to tree-based approaches.

## 1. Introduction

Location-Based Services (LBSs) have been extensively used in outdoor environments by several applications due to the ready availability of accurate positioning information [1,2]. Currently, most mobile devices are equipped with Global Positioning System (GPS), allowing the development and growing usage of some exciting and useful applications such as Waze and Google Maps, to cite a few [3].

Unfortunately, even though several LBSs have been used in outdoor environments, the same cannot be observed in indoor settings, such as offices, shopping malls, and parking lots. The main reason for this is the inaccurate positions in these environments reported by GPS since the latter requires a direct view of the satellites to work properly [4]. Thus, many interesting applications that could exist today still have not been developed for these indoor scenarios due to the lack of accurate positioning technology.

Currently, the leading available solution to this problem is the Indoor Positioning Systems (IPSs). In this case, a local infrastructure available or installed in the building is used to allow the precise location of mobile devices inside this building. Among the most used infrastructure for IPS is WiFi, due to its abundant availability and no need for additional hardware, since most buildings and devices (e.g., smartphones, smart TVs, Internet of Things) already come equipped with this technology. In this case, the Received Signal Strength Indicator (RSSI) of the packets received by the WiFi access points is used to locate the mobile device that sent these packets.

However, using the available WiFi infrastructure for an indoor location of mobile devices is not something trivial due to the high variability of the RSSI [5]. Unfortunately, the several obstacles and walls inside buildings make it difficult to translate a signal strength into distance information to be used in the positioning. To overcome this problem, currently, the most considered solution for an IPS is using the fingerprint technique.

**Figure 1:**
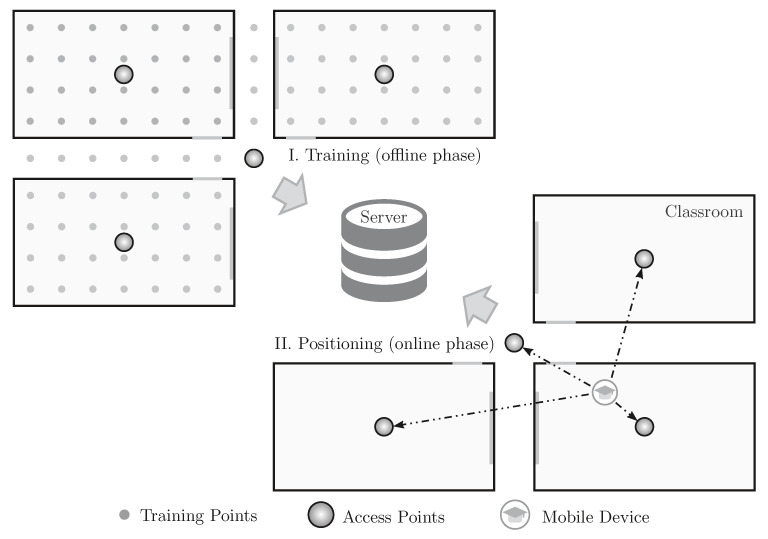
The two phases of a centralized fingerprint-based indoor positioning system implemented in a campus scenario: training and positioning.

As depicted in Figure 1, the fingerprint technique is divided into two phases: training and the positioning, also known as offline and online phases, respectively. In the training phase, several points of the scenario are trained by physically going to these locations and sending WiFi packets. The packets sent from each training point are received by several access points that can compute the RSSI of these packets. All of this information is then sent to a central server that stores them in a training database. In the positioning phase, which is when the IPS is online and working, when a mobile device sends a packet, the RSSIs of this packet perceived by several access points are sent to the central server. The server then compares the received RSSIs to the ones stored in the training database to find the training point that mostly matches the received data. The returned point is the position of the mobile device.

**Figure 2:**
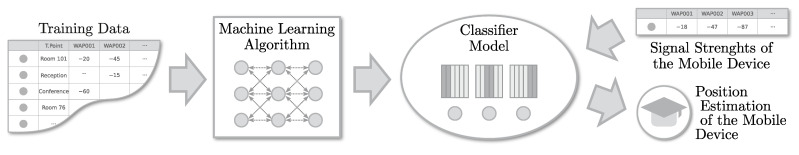
A typical architecture for a fingerprint-based indoor positioning system running on a centralized server.

To implement a fingerprint-based IPS, we typically use an architecture as depicted in Figure 2. Usually, supervised machine learning techniques are among the most viable solutions [6,7]. They use the previously recorded training database to train a classifier, such as the k-Nearest Neighbors (kNN) [7,8,9], so it can be used to estimate/classify the positions of the nodes later in the online phase.

The use of the fingerprint technique for this purpose has grown due to two crucial factors: (1) there is no need for additional hardware to gather location information since the WiFi infrastructure is usually enough; and (2) the ability of the system to remain consistent even with the unpredictability of the signal strength values of the devices. Recently, the fingerprint technique has been improved by several proposals; in some works it is already possible to locate devices in an indoor environment with errors of only one meter or so [6,8,10].

kNN is one of the most used classification methods in fingerprint-based positioning systems due to its high-precision results [11]. However, it lacks scalability since each sample to be classified must be compared to all other samples in the training database [12,13]. Furthermore, the training database tends to increase in size drastically as we increase the location scenario or when we need more precision, since the more data we have, the more precisely it tends to estimate the positions [14].

If we take an example of a medium-sized room where there may be several training points (e.g., one for every two square meters), and knowing that for each of these points we need several packet samples (e.g., between 100 and 1000 packets), we can quickly have more than tens of thousands of samples in the training database collected from a single room. Moreover, each sample (row) in the database has several attributes (columns) according to the number of WiFi access points present in the whole scenario. Thus, it is easy to see that as we increase the area of the IPS, we also need to add more access points, increasing both the number of attributes and the dimension of the classification [6]. Taking the example of a shopping mall and a university campus, which are relatively large in scale, the database used for training and positioning can easily reach billions of samples with hundreds or even thousands of attributes. Finally, taking into account that the goal is to have real-time location information for each of the thousands of mobile devices every second (something easily done by GPS), it is clear that a highly efficient classification method is required [10,12,15].

Thus, to achieve a highly efficient IPS, in this work we propose the use of a novel technique called Hierarchical Navigable Small World (HNSW) [16]. In this technique, a graph-based data structure is carefully crafted to have the characteristics of a small-world network [17]. This data structure is then used to find the nearest neighbor, as done by the kNN technique.

We evaluated the performance of the solution using our proposed large-scale synthetic fingerprint dataset generator. We argue and show that this simulation-based synthetic dataset can evaluate the performance of any large-scale IPS under several different scenarios, something not possible to do in a real-world testbed. Additionally, we also carried out high-scale real-world experiments, implementing a complete IPS testbed in a school building. Finally, we compared our results to the UJI Indoor dataset, used by several known works to evaluate the performance of IPSs [18]. In all of these cases, we compared the performance of the HNSW to two other known kNN optimizations: Kd-Tree and Ball-Tree. Our results clearly show the performance gains when using the graph-based solution by 98% when compared to classic kNN and at least 80% when compared to the tree-based approaches.

This work is an extension of our previous work presented in [4]. We improved our solution and significantly extended the performance evaluation by creating a new IPS testbed implemented in a school building composed of 60 different rooms covered by 42 access points. We trained most of the school area with 488 different training points, averaging 2000 samples per point to a total of 1,020,446 samples in our dataset.

The remainder of this paper is organized as follows. In the next section, we present our related work. The graph-based technique is explained in Section 3. We then show and discuss our performance evaluation in Section 4. Finally, in Section 5, we discuss the applicability of the proposed solution, and in Section 6, we present our conclusions and future work.

## 2. Related Work

Our related work is divided into three categories: (1) scenario analysis and fingerprint, in which we show known fingerprint-based techniques; (2) computational cost reduction, in which we show proposed techniques to improve the efficiency of the fingerprint, usually reducing the training data; and (3) efficiency without loss of information, in which we show some current work that is more closely related to our approach that improves efficiency without reducing the training data.

### 2.1. Scenario Analysis and Fingerprint

The work developed in [19] was one of the first to use the RSSI of WiFi devices as information to estimate their locations. The proposed fingerprint-based solution, called RADAR, resulted in an accuracy of 2 to 3 m. Subsequently, as seen in [20], the authors have made improvements based on the Viterbi algorithm, increasing the accuracy of location up to 33%.

Roos et al. [21] presented a fingerprint-based approach to estimate the locations of users. The authors considered the location as a machine learning problem, where the task would be to model how the RSSIs are distributed in different geographic areas based on samples collected in various known areas. From this, they presented a probabilistic structure responsible for estimating the location of the mobile devices. Such a structure uses Bayes’ theorem to compute the likelihood between the collected signals given each possible location for the same. Using real-world samples to demonstrate their results, they have shown estimates with an average location error of about 2 m.

In [22], a probabilistic approach is also proposed. The authors collected the data received throughout a three-story building, remaining about 2 min in each office or region with a mobile device emitting the signals, resulting in a location accuracy of 95% in all predictions. However, the proposed solution has a room-based resolution, which is not able to pinpoint the position of the users inside the rooms. The results of this work also presented robustness concerning various unforeseen phenomena at the time of data collection, such as the presence or absence of people in the environment throughout the day. These and most current proposed solutions focus mainly on the accuracy and precision of the systems [11], while other aspects related to complexity and performance are ignored.

Geometrical, model-based approaches, such as multilateration and trilateration, analyze the scenario characteristics to create a propagation model that converts signal strengths to distances. They are very efficient in this regard since no searches in datasets are required. However, they have lower accuracy [13,23]. Similarly, probability-based approaches are usually more accurate than geometrical ones, but they are still not as precise as fingerprint-based methods for larger and more complex environments [24,25].

Finally, computer vision techniques have also been proposed to improve IPSs in various ways [26]. It is possibly the most viable solution to provide fine-grained tracking capabilities to IPSs due to the high variability of the RSSI and other sensors used for positioning. In [27], the authors combine the coarse, fingerprint-based IPS with computer vision analysis from cameras in the scenario to provide a wide-area, fine-grained IPS. On the other hand, in [28], the authors use a mobile, camera-enabled device to provide a computer vision-based indoor navigation system to help blind and visually impaired people, an application that requires high accuracy.

### 2.2. Computational Cost Reduction

To reduce the computational cost, ref. [15] proposed a technique based on clustering, in which it groups the locations from a set of WiFi access points. Called Joint Clustering, the technique has two key points: (1) use of probability distributions to improve accuracy and (2) use of groups of mapped data to reduce the cost of online positioning. Later, in [29], the authors presented the system entitled Horus, following the same probabilistic approach and optimization from clustering, but with advances in the preprocessing of signal power models to consider spatial and temporal variations characteristics of the wireless channel. The results of the Horus system presented an average precision of up to 39 cm in one of the tests performed.

Chen et al. [14] seek to reduce the computational cost by reducing the size of the database used to train the classification algorithms. They developed an algorithm called CaDet that selectively chooses some access points to be used in the estimation, allowing an intelligent subdivision of access points necessary for a precise location. In this way, by reducing the required number of access points for each estimate, there is an efficiency gain. Furthermore, the solution allows for a lower energy cost, which is paramount to the approach, since the classification processing is done directly on mobile devices. CaDet uses a combination of information theory, cluster analysis, and decision tree to achieve good accuracy with performance gain.

In [7], Yim et al. sought to optimize IPSs by proposing the discretization of the RSSI values in the database to train a decision tree-based model during the offline phase. The authors had the premise of reducing the time needed to both generate the training model and locate the mobile device. From that, they presented experiments that demonstrate gains concerning the complexity of the proposed approach. However, little was said about the results of accuracy, only making it clear that they are no worse than the methods compared.

Fang et al. [6] reduced the computational cost by combining related access points information and using Principal Component Analysis (PCA) to decrease the dimensionality of the data. An algorithm is proposed to intelligently transform incoming signals into main components, instead of selecting access points, so the information received can be used more efficiently, reducing the computation required in the online phase. The authors have demonstrated that this solution, compared to other approaches, can reduce the positioning error by 34% and has a 40% reduction in time complexity. A similar PCA-based approach was proposed by Salamah et al. [30]. Furthermore, the transformation into main components results in some other benefits, such as the need for fewer training data and tolerance to anomalies in the received signals.

More recently, the problem of computational cost reduction in fingerprint-based IPSs has been overcome by employing other novel clustering and graph-based techniques. However, the reduction in the computational load often comes with accuracy issues [31]. These advances of the area resulted in different approaches concerning traditional clustering such as *K*-means [32], hierarchical [33,34], and other novel clustering techniques [35]. Graph-based data structures have also been proposed recently to improve IPSs. In [36], graphs are used to connect neighboring scenario landmarks to improve accuracy and computational resource when using spatial information. In [37], the authors propose the use of graph-based semi-supervised learning (G-SSL) to reduce and spatially average the RSSI values to improve accuracy while smoothing the fingerprint dataset.

While all of these works result in the reduction of the computational cost, most of them are based on the removal or combination of features from the training base, resulting in the loss of information. This information removal can lead to higher errors in specific scenarios since there is usually a loss of potentially relevant information, also making it difficult or even impossible to implement adaptive solutions and other forms of calibration, which can be used to reduce the location errors [38].

### 2.3. Efficiency without Loss of Information

In our current proposal, we focus mainly on solutions that do not need to lose training information to achieve better performance. Some known algorithms of tree-based classification have been proposed with this goal, such as Ball-Tree [39] and KD-Tree [40], as well as other approaches [7,14,41,42] that take advantage of this type of data structure to quickly search and find, within the training base, a set of data that behaves similarly to the search entry. Recently, the use of graph-based methods to search for the *k*-nearest neighbors have shown competitive gains in performance when dealing with large datasets, as seen in [43,44].

Recently, compression techniques have also been applied to datasets to improve kNN [45]. In [46], for instance, the kNN-based training dataset is compressed so that larger datasets can fit in the memory. An interesting aspect of this work is that a sample is decompressed in real-time (on-the-fly), without needing to decompress the whole dataset. In this case, even though the dataset size is reduced, no information is really lost.

Thus, in this work we seek to explore the application of hierarchical navigable small world graphs, recently proposed by Malkov et al. [16], to reduce the computational cost of IPS without loss of information while maintaining the accuracy of the results. The main idea of this algorithm is to use graph-based data structures and small world techniques to improve the search for areas that are similar to the input entry. The use of small world techniques allows large jumps in the graph, as explained in more detail in the next section. As far as we know, this is the first time that a graph-based machine learning algorithm is explored and applied to indoor positioning systems.

## 3. IPS Using Hierarchical Navigable Small World Graphs

When it comes to optimizing the search for a particular area of the training database where the attributes are similar to the one we are searching (i.e., the nearest neighbors), most approaches tend to perform a reduction in the training data, causing a loss of information [7,14,42] or are based on a tree structure to rearrange the training data [39,40,41].

Recently, some proposed solutions have shown significant advances in the use of graph-based methods to search for the nearest neighbors on high-dimensional datasets. This efficiency in high dimensional datasets is an essential aspect for fingerprint-based high-scale indoor positioning systems since a training database can be composed of hundreds to millions of access points (i.e., attributes, dimensions).

One of the critical points for the use of a graph-based structure is the implementation of a small world model. In this case, the generated graph has two essential characteristics for the search process: (1) small path length values between nodes (samples in the case of IPSs) and (2) high-cluster coefficient values [17]. The first feature allows for a quick (logarithmic or less) searching for a similar sample in the training database. This fast search happens because a small percentage of distant nodes can have links between them, allowing the search to bypass most of the database in a single hop. The second feature guarantees the connectivity of the nodes (existence of paths) and facilitates the location of the *k* values closest to a given sample.

Based on that, a proximity graph for the nearest neighbor search called Navigable Small World was proposed in [44,47]. These solutions use an alternative approach of the simple greedy search used on proximity graphs and also a probabilistic skip list structure [48] that allows a logarithmic scaling for the search.

Proposed in [16], the Hierarchical Navigable Small World Graphs (HNSW) implemented the idea of representing each sample of the dataset as a node in the graph. It separates their links according to their length scale, producing a multilayer graph that allows the evaluation of only a small subset of the connections for each element, thus reducing the computational cost of performing the search.

**Figure 3:**
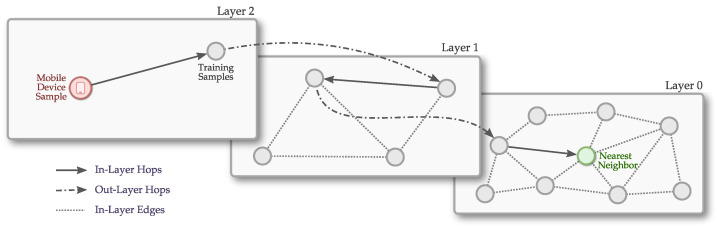
Indoor positioning system classification using hierarchical navigable small world graphs.

To build the multilayer graph, the links are set with different distance scales by artificially introducing layers, as depicted in Figure 3. Each layer is represented by an integer number, called level number, and every node on the graph receives a level number representing the maximum layer to which this node belongs. The top layers represent connections between distant nodes, which gives the graph its main small-world characteristics. On the bottom layers, the graph has the connections between closer nodes, connecting a node to its neighborhood. To facilitate understanding, Algorithm 1 shows an algorithmic overview description of the functions for creating and searching the multilayer graph. More details on the workings of the solution, as well as a complete set of algorithms, can be found in [16]. Each node is inserted on the graph (line 7) at the layer represented by its level number, as well as at the descending layers up until the ground layer (Layer 0), being linked to its nearest nodes in each level (lines 18–23). The level number is randomly selected, and an exponentially decaying probability distribution is used to concentrate the nodes at the lower layers and enable close connections, and letting only a few nodes at the top layers, using a parameter to normalize this distribution. This step is shown in line 8 of Algorithm 1.
**Algorithm 1:** HNSW graph generation and search overview.▷ **Global Variables:** 1: *trainingDataset*;{Complete KNN training dataset} 2: *hnswGraph*;{HNSW multilayer graph} 3: *topLayer*;{Maximum, top layer of the *hnswGraph*} 4: *entryPoint*;{Entry point for the *hnswGraph*} 5: *mL*;{Normalization factor for level definition} 6: *ef*;{Number of nearest neighbors to use}▷ **Function:** 7: *hnswGraphGeneration*(){Generate *hnswGraph* from *trainingDataset*}
 **Action:**
 8: **for** each element *newElement* from t*rainingDataset* **do** 9:    *maxLayer* ← ⌊−*ln*(*uniform*(0 ... 1)) × *mL*⌋;{Compute the maximum layer of the element}10:    **if** *entryPoint* = = *nil* **then**{First inserted element.}11:      *topLayer* ← *maxLayer*;12:      *entryPoint* ← *newElement*;{Sets the entry point for the *hnswGraph*}13:      **next**;14:    **end if**        {Search the nearestNode already in *hnswGraph* starting from the *topLayer* until *maxLayer*}15:    *nearestNodes* = *entryPoint*;16:    **for** *currentLayer* ← *topLayer* ... *maxLayer* **do**17:      *nearestNodes* ← *searchLayer*(*newElement, nearestNodes, currentLayer*);18:    **end for**        {Insert the newElement in all of the layers from maxLayer to layer 0}19:    **for** *currentLayer* ← *maxLayer* ... 0 **do**20:      *nearestNodes* ← *searchLayer*(*newElement, nearestNodes, currentLayer*);21:      *neighbors* ← *selecNeighbors*(*newElement, nearestNodes, currentLayer*);22:      *insertElement*(*newElement, currentLayer, neighbors*);23:    **end for**24:    **if** *maxLayer* > *topLayer* **then**25:      *topLayer* ← *maxLayer*;26:      *entryPoint* ← *newElement*;{Set entry point for *hnswGraph* to *newElement*}27:    **end if**28: **end for**▷ **Function:**29: *hnswSearch*(*element*){Search nearest nodes for *element* from *trainingDataset*}**Action:**30: {Search the element in *hnswGraph* starting from the *topLayer* until 0}31: *nearestNodes* = *entryPoint*;32: **for** *currentLayer* ← *topLayer* . . . 0 **do**33:    *nearestNodes* ← *searchLayer*(*newElement, currentLayer, nearestNodes*);34: **end for**35: **return** *nearestNodes*;▷ **Function:**36: *searchLayer*(*element, nearestNodes, layer*){Search the *ef* nearest nodes for *element* in *layer*}37:{using *nearestNodes* as enter points.}**Action:**38: *nearestNodes* ← {Greedy graph search algorithm as described in [16]}39: **return** *nearestNodes*;

After the graph construction, as depicted in Figure 3, when a new search is required, it starts on the highest layer (line 32) and greedily traverses the graph, selecting elements only at that layer until it finds a local optimum. Once the local optimum is found, the search continues on the next layer below it in the hierarchy. This process is repeated starting from the local minimum found in the previous layer, using its nodes as entry points, and so on until the ground layer is reached. These steps are shown in lines 29–35 of Algorithm 1.

A variant of a greedy search algorithm based on [44] is used to find the closest neighbors at each layer. A dynamic list is used to keep the *ef* closest nodes found at that layer, and during the search, this list is updated at each step by evaluating the neighborhood of the closest previously non-evaluated element in the list until the neighborhood of every element from the list is evaluated. Initially started as 1, to represent a simple greedy search, the *ef* parameter is incremented up to *efConstruction*, another parameter that is used to control the recall of the greedy search procedure. This value can be increased to improve the quality of the constructed graph, leading to higher accuracies but also increasing the indexing time. Another parameter, *M*, defines the maximum number of neighbors in the zero and above-zero layers. In most of our experiments, we used the default value of 100 for both the *efConstruction* and *ef* parameters and the value 15 for *M*, which resulted in the best accuracies. Since the presented algorithm is an overview, for the sake of simplicity, we did not detail these steps, but they can be found in [16].

The HNSW has two of the main characteristics we need for a fingerprint-based, high-scale IPS: (1) connectivity (clusterization) of near/similar samples, allowing the election of the best sample based on the *k* nearest neighbors (kNN), and (2) a fast search for the sample with most similar attributes. In this work, we explore the use of the HNSW in the task of estimating the location of mobile devices in a fingerprint-based IPS. Our performance evaluation methodology, as well as our obtained results, are discussed in the next sections.

## 4. Performance Evaluation

Our performance evaluation aimed at comparing the cost of building the search index (model fitting) and also the cost of searching for the nearest neighbors (sample classification) achieved by the studied methods. Furthermore, we analyzed the error of the indoor positioning system when varying the algorithm adopted to find the nearest neighbors. Below we describe our evaluation setup and experimental results.

### 4.1. Methodology and Datasets

We compared the hierarchical navigable small world graphs (HNSW) to two tree-based methods: Kd-Tree and Ball-Tree. We also compared the results to the classic kNN search, commonly known as brute force. For the implementation of the HNSW search, we used the Non-Metric Space Library (NMSLIB) [49], while for the other methods we used the Scikit-Learn [50], both using Python language. All of the results were reached through the mean of a ten-fold cross-validation [51].

We evaluated the performance of the implemented solutions using three very different datasets:

1. Synthetic Indoor Positioning Dataset
2. SmartCampus Indoor Positioning Dataset
3. UJI Indoor Localization Dataset

These datasets are detailed in the next sections.

#### 4.1.1. Synthetic Indoor Positioning Dataset

We built an indoor positioning simulator based on known propagation models for indoor environments to generate synthetic IPS training databases from different scenarios such as varying the number of routers, rooms, and training points. By using this simulator, we were able to evaluate the performance of the studied algorithms in hundreds of different indoor scenarios, something that would not be feasible to do in a real-world testbed. Most of our results are based on this simulator.

Even though we implemented some of the most known propagation models such as Free-Space [52] and Log-Distance [53], they did not result in a good performance for the positioning since they do not take into consideration some scenario information such as walls, floors, and ceilings. Thus, we implemented our model based on the known Simple Indoor Signal Propagation Model [54], in which the authors proposed a heuristic indoor path loss prediction model that is both simple and quick (as the statistical models) but is also very accurate. They formulated an indoor path loss model for 2.4 GHz-band, performing simulations in four very different buildings to help on the path loss measurements and parameter determinations. The model takes into consideration both wall and floor attenuation factors, as suggested in [19].

**Figure 4:**
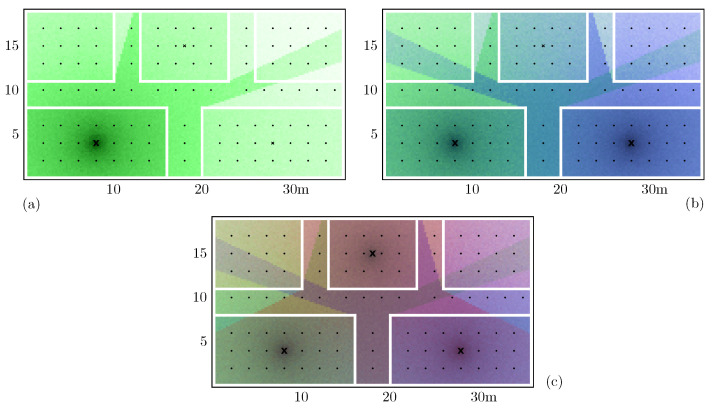
Example of a signal strength simulation: white lines are the walls of the rooms. X marks the position of the routers. In (**a**), we have only a single router in the most bottom-left room, while in (**b**,**c**) we have two and three routers, respectively.

By feeding the simulator with the map of the location, as well as the values for the model variables, we were able to compute the RSSI perceived by the routers from any place in the scenario. Figure 4 shows an example of the generated signals strengths in a simple scenario composed of five rooms. In Figure 4a, we have only a single router in the most bottom-left room, while in Figure 4b,c, we have two and three routers, respectively. From this, to create our synthetic positioning training dataset, we obtained several samples of RSSIs from equally spaced training points inside the rooms.

The synthetic training dataset generated by us has 378 distinct rooms within one floor, including 196 that simulate rooms and 182 that simulate halls. There are 21 reference points, uniformly spaced and 2 m apart, for each room and 7 in each hall, where we simulated 50 samples for each point, for a total of about 270,000 samples.

#### 4.1.2. SmartCampus Indoor Positioning Dataset

The second dataset used in our performance evaluation was based on our real-world indoor positioning testbed. We implemented a complete Bluetooth Low Energy (BLE) IPS in a working high school composed of 42 custom-made routers (we called them scanners) and 60 rooms. For each room, we trained several training points every two meters. For each training point, we collected more than 2000 samples, resulting in an indoor positioning dataset composed of 1,020,446 samples. Regarding the mobile devices (beacons) used for data collection, we used 55 BLE-enabled, smartwatch-like devices, each sending one advertisement packet per second. Thus, a single reference point was trained in about 2–3 min, which is important when considering the 488 reference points that needed to be trained. The whole school was trained in about 5 days.

This testbed is the beginning of a bigger project to create an indoor positioning system financed by Positivo Technologies, one of the biggest technology companies in Brazil. This project, called SmartCampus, aims at providing location information of students in high schools, allowing several interesting applications such as:

- Automatic student attendance record keeping;
- Checking whether a student is in the classroom he is supposed to be;
- Entry and exit control of students from the school;
- Where the student sits inside the classroom;
- Interaction among students and friend groups.

**Figure 5:**
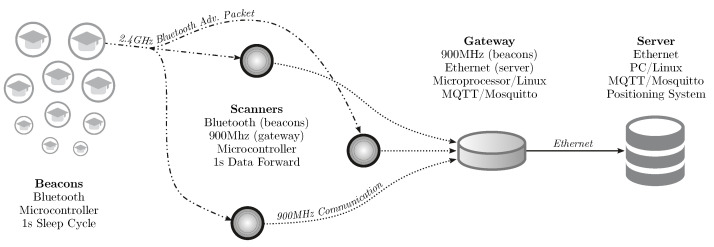
SmartCampus Architecture: a complete, large-scale, indoor positioning system testbed. All beacons send a Bluetooth advertisement packet every second. These packets are received by the scanners, which forward the data to the gateway using a 900 MHz, long-range, communication. The gateway sends the data to the central server, which can localize all of the beacons using the proposed graph-based technique.

For this, we proposed and implemented the SmartCampus Architecture, depicted in Figure 5. In this architecture, beacon nodes (worn by the students) send BLE advertisement packets [13,55]. These packets are received by several Bluetooth-enabled devices, called scanners (similar to routers in WiFi IPSs). These scanners compute the RSSI of the received packets and send them directly to a central device, the gateway, using long-range, 900 MHz, communication. The gateway is connected to a central server, which can locate all of the received beacons using the proposed graph-based machine learning technique.

The main reason for using a BLE-based architecture is the current restrictions imposed on smartphones and other devices to limit the access to WiFi RSSI information due to energy and privacy concerns. For instance, both iOS and Android-based devices have these restrictions that mostly prevent the implementation of any fingerprint-based system. In contrast, BLE-based LBSs have seen an increasing interest in new and novel applications such as AirTag [56] and Tile [57]. Finally, since the focus of our work is on the fingerprint technique, we did not implement other infrastructure-free techniques such as magnetic fields or inertial-based systems that could be used to improve positioning.

**Figure 6:**
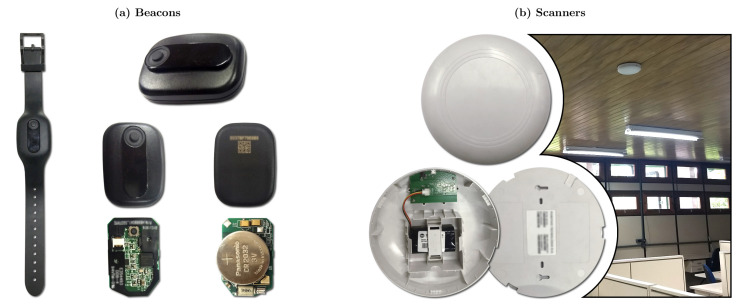
SmartCampus Hardware: (**a**) beacons with Bluetooth communication; one beacon is inside a bracelet, as used by the users; the other beacons are outside the bracelet (front and back), and opened to show hardware (front and back); and (**b**) scanners with Bluetooth and 900 MHz long-range communication (front, opened, back, and installed on the ceiling). The gateway has a similar hardware footprint as of the scanners, and the server is not shown.

**Figure 7:**
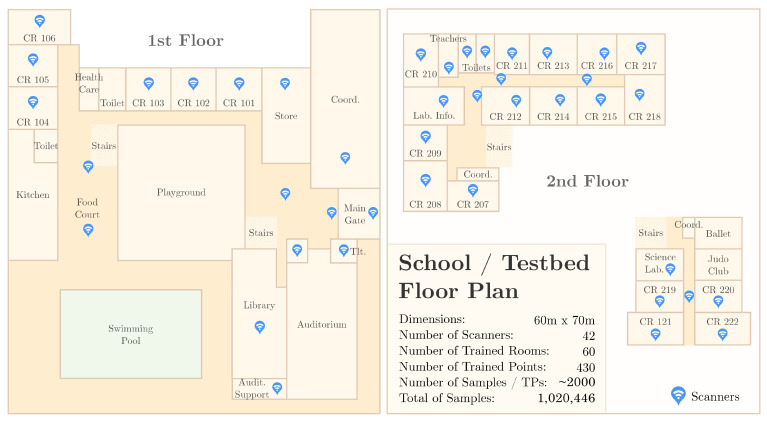
SmartCampus Testbed Floor Plan: an entire school composed of 60 rooms/halls were trained. Forty-two scanners were distributed throughout the school; 488 points were trained, about 2 m distant from each other; for each training point, about 2000 samples were collected, to a total of 1,020,446 samples.

All of the hardware used in the SmartCampus architecture was developed and created by Positivo Technologies, which is part of the team for this current work. The hardware is depicted in Figure 6. Finally, Figure 7 shows an overview of the floorplan of the school in which we implemented the testbed as well as the location of the gateways.

#### 4.1.3. UJI Indoor Localization Dataset

For the third dataset used to evaluate the performance of the techniques, we used another real-world scenario: the popular UJI Indoor Localization dataset [18]. This dataset has been used in the literature and allows the comparison of our results to other published work. Furthermore, this scenario is quite different from our SmartCampus testbed. It covers a surface of 108,703 m^2^ with three different buildings comprising 4 to 5 floors. There is a total of 520 routers with a total of 21,049 samples. The data were collected by more than 20 users with 25 different mobile device models.

Compared to our SmartCampus dataset, it is easy to see that the UJI testbed scales on the number of routers since it has 520 routers while our testbed contains only 42 routers. However, the UJI dataset does not have several samples per training point, and these training points are not equally distributed. Thus, the UJI contains only 21,049 packet samples, while our SmartCampus testbed contains more than 1 million samples. Therefore, our SmartCampus testbed scales on the number of samples.

Thus, besides the already mentioned popularity of this dataset, as well as its higher number of routers, another important aspect of this dataset is its lower scale. Having only 10 samples per reference point on average, this dataset allows us to understand the behavior of the solution in similar real-world cases, that is, smartphone and WiFi-based applications where it is difficult to collect a large number of samples per reference point due to the limitations imposed by these devices.

To summarize all of these aspects, Table 1 compares the main characteristics of the three datasets. In the next section, we show the results of our performance evaluation, starting with the experiments with the synthetic base, since this is the most controlled scenario.

### 4.2. Impact of the Number of Samples on the Classification

The use of a graph-based data structure by the HNSW is done to reduce the search time required at the time of classification and also to avoid an increase in the positioning error. With this in mind, in our first experiment, we varied the number of samples in the training database from 54 thousand to 270 thousand samples to analyze the scalability of the methods as the size of the training base increases. In this scenario, we had 196 access points (attributes in the dataset).

**Figure 8:**
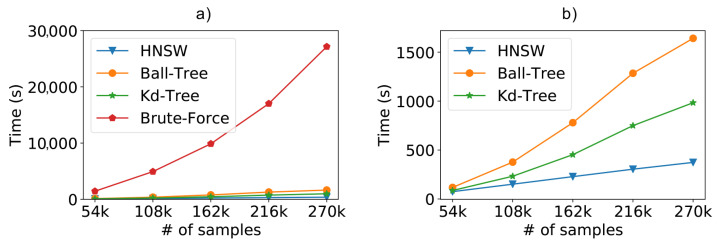
Scalability test results from a dataset containing 196 access points (attributes) and varying the number of samples. (**a**) Total time including the brute force and (**b**) without the brute force method.

For the synthetic database scenario, we can see in Figure 8a the results regarding the performance of the compared methods. In this case, we are comparing the time needed to classify 10% of the database (used as the test dataset), using the ten-fold cross-validation. It is easy to see that any of the optimized methods exhibit a considerable performance gain over the classic kNN using brute force.

To facilitate the visualization, Figure 8b repeats the previous experiment without the brute force kNN, since the difference from the optimized methods is too high. Now, we can clearly see that the HNSW technique is also able to outperform the tree-based approaches. In these experiments, the graph-based technique was about 2.5 times faster than Kd-Tree and 4 times faster than Ball-Tree. Furthermore, we can easily see the difference in the curve behavior. For now on, we will refrain from showing the brute force kNN in order to better evaluate the optimized approaches.

**Figure 9:**
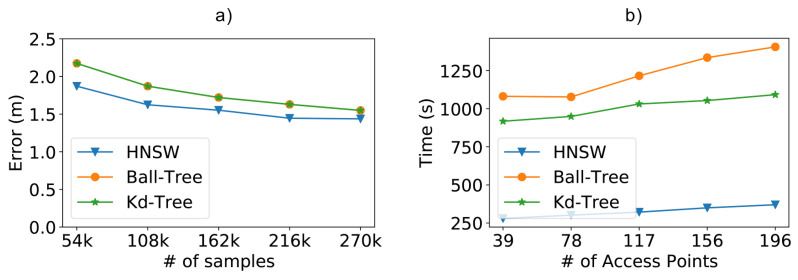
Scalability test results from a dataset containing 196 access points (attributes) and varying the number of samples. (**a**) Positioning error obtained by the experimented methods; (**b**) impact of the number of access points on classify time.

In Figure 9a, we compare the results of the methods concerning the positioning error as we increase the size of the training database. As expected, the more training data we have, the lower the positioning error. In our case, we can see that the position error decreased from 2.17 m to 1.43 m, a reduction of 34%, as we increased the number of samples from 54,000 to 270,000. These results help us realize the importance of additional samples in the training database for more accurate positioning. Although the brute force method is not on the chart, it is important to note that it achieved roughly the same error results as Kd-Tree and Ball-Tree. In the chart we can also notice another significant result: the HNSW technique resulted in smaller positioning errors when compared to tree-based methods. Even though the difference is not much, it was still possible to reduce the positioning error by 3% at best.

### 4.3. Impact of the Number of Access Points

The number of access points is also another factor that significantly affects the computational cost of an IPS since each additional access point is accountable for including a new column (attribute) for all samples in the training database. In addition, increasing the density of the access points is also a known technique used in the literature to reduce the positioning error, where better accuracy is sought by increasing the number of access points per square meter.

To assess this impact, we varied the number of access points in the experiments from 39 to 196, but we kept the density of the access points constant; i.e., we increased both the number of access points and the size of the scenario (number of rooms and number of training points). This constant access point density is needed, since we wanted to analyze the impact on the computational cost but without the positioning error being affected by poor distribution of the density of the access points.

As depicted in Figure 9b, another interesting aspect of the HNSW technique is that its performance advantages also scale up as we increase the number of access points, being 74% faster than Ball-Tree for 196 access points.

### 4.4. Impact of the Number of Samples on the Model Fitting

One of the steps in an IPS architecture is the creation of the model or the indexing of the data in its respective search structure, based on the available training data (the fitting task). This step was depicted earlier in Figure 2. Even though the fitting task execution time is not an essential factor in IPSs since it is executed only once, it might affect some known techniques that require a periodic model fitting, such as calibration-based and adaptive solutions [38] that are known to reduce the positioning error over time even more.

**Figure 10:**
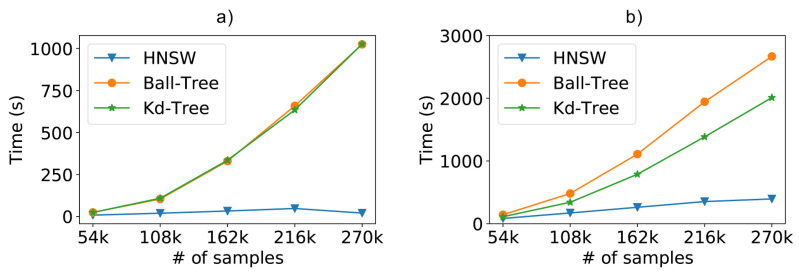
Scalability evaluation including the (**a**) model fitting time; (**b**) the total time (fit+classification).

In this way, Figure 10a depicts the fitting times as we increase the number of samples in the training database. For this task, the brute-force method (not included in the chart) has a constant time close to zero, since it does not execute any indexing of the data. As we can see, especially in the larger scenarios, the creation of the graph-based model is faster than the tree-based models, reaching 98% faster fitting times.

Finally, Figure 10b shows the complete total time (fitting+classification) of the tested models. When including both model creation and sample classification, the HNSW technique is shown to be 85% faster than Ball-Tree and 80% faster than Kd-Tree.

### 4.5. SmartCampus Dataset Experiments

As mentioned before, we also performed real-world experiments of our solution by implementing a complete IPS in a school building. The resulting SmartCampus dataset represents a real-world scenario in a building with distinct floors and spaces, and it approaches the number of samples of the synthetic dataset, allowing the analysis of the scalability of our proposal in a real scenario. Despite this, the SmartCampus is the dataset with the lowest of access points among the three evaluated datasets, even though 42 is a reasonable amount of access points (higher than most experiments in the literature) and is also wholly consistent with many situations in the real world.

**Figure 11:**
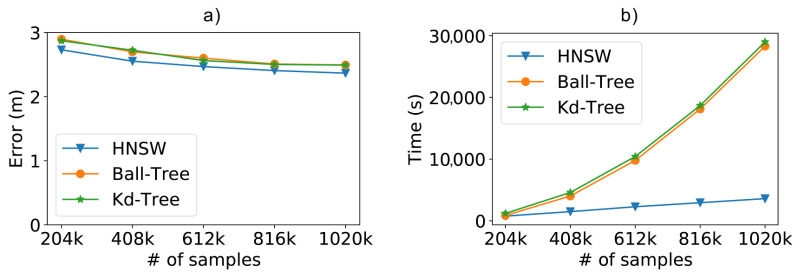
(**a**) Positioning error and (**b**) total time (fitting + classification) when using the real-world SmartCampus dataset and increasing the number of samples with K=3.

With the complete dataset, we analyzed the impact of the number of instances, to train and classify, on precision and time. In Figure 11a, we can see that there was a slight advantage in the use of graphs, with a mean error difference of a few centimeters, when looking to the best results achieved for each method.

For this experiment, we changed the default values of the HNSW parameter *M*, from 15 to 40, and the parameter *ef*, from 100 to 150. In other words, we allow greater connectivity in the graph and allow a search with greater recall, respectively. This adjustment was necessary to reach the accuracy comparable with the other methods, with up to a certain advantage, which meant an extra cost of performance, compared to the standard values of the parameters. However, the method allows this flexibility, and yet we maintained a remarkable superior performance. It is important to note that these values were chosen based on preliminary analyses using the ten-fold cross-validation technique [51], which can be used to find these parameters on any real-world dataset.

Besides showing the advantage of the proposed method, Figure 11a also confirms the impact of the amount of training samples on system accuracy. Furthermore, as we increase the number of data to achieve better results, the performance gain achieved by HNSW is impressive, the total time taken by it to fit and classify the entire SmartCampus dataset was about 8 times faster than the other compared methods, as can be seen in Figure 11b.

**Figure 12:**
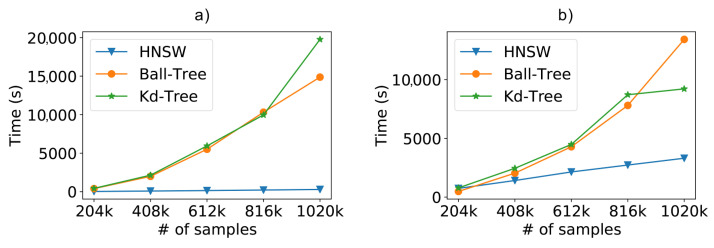
(**a**) Fitting time and (**b**) classification time evaluation results when using the real-world SmartCampus dataset and increasing the number of samples with K=3.

Detailing each different task, the graph-based method still shows advantage in both steps. We see in Figure 12a that there is not much variation in fit time as we increase the number of training instances, since the construction of the graph takes a constant time. Furthermore, even though all methods have comparable classification costs when it comes to a few instances, as shown in Figure 12b, so the huge difference in graph performance presents itself as we increase the number of samples.

### 4.6. UJI Dataset Experiments

Finally, we also performed our experiments using the popular UJI Indoor Localization [18] dataset in order to evaluate the performance of the methods using real scenario data available in the literature. A point to take into account with this training dataset is that even though it has 529 access points (attributes), it only has approximately 20,000 samples, since for each place determined for the collection of the samples, there was little to no repetition; that is, only a few packets were sent by the mobile device from each training point.

For this reason, the training dataset of the UJI has only 10% the number of samples from our synthetic database. On the other hand, this dataset has 2.7 times the number of access points, which is an interesting point to analyze, since it is a very different case from our previous experiments. Since this is a relatively small dataset, and since we did not have much control over it, we kept all of the fixed scenario characteristics and changed the value kNN *k* from 1 to 11 to perform the experiments.

**Figure 13:**
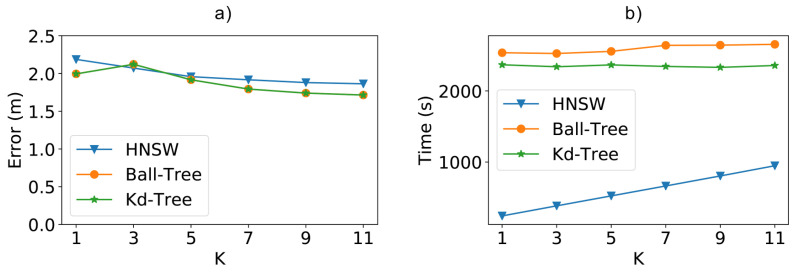
(**a**) Positioning error and (**b**) total time (fitting + classification) when using the real-world UJI indoor dataset and varying the *k* value.

Analyzing the results in Figure 13b shows that the HNSW method was faster in all cases, being 90% faster than Ball-Tree and 89% faster than Kd-Tree for *k* equal to 1. In the figure, we can also see that the total time for HNSW increases at a higher rate than the other tree-based methods, showing the influence of the *k* value on the creation of the graph structure. Thus, for even higher values of *k*, it is possible for the total time of HNSW to be closer to the other techniques. However, as we will see in the next paragraph, the positioning error does not decrease accordingly. For instance, increasing the value of *k* from 9 to 11 increased the total time by 20%, while making almost no difference in the positioning error.

Regarding this positioning error, as depicted in Figure 13a, while we increase the value of *k*, the error begins to fall and soon tends to remain stable. Thus, the lowest *k* values already reach the best results for the techniques, especially when considering the required extra computation cost for higher values. Furthermore, despite the small disadvantage of a few centimeters in the best results, the method still remains competitive with the large performance gain in cases of large access points as well.

When analyzing the behavior of the *k* value in terms of both time and error, we can see that a value of 7, in this case, would result in the best compromise. For this value of *k*, the time for HNSW is 76% faster than Kd-Tree and 79% faster than Ball-Tree, with a slight increase of 0.11 m on the average positioning error.

## 5. Applicability of the Proposed Solution

Despite the good results obtained in our performance evaluation, we understand that these results are indications that the proposed solution can be used only in some scenarios, but not all of them. For instance, we did not evaluate the performance of the solution when combined with other known techniques that improve accuracy such as crowdsourcing or computer vision.

However, we tried to implement the solution in very different scenarios. For instance, the UJI Indoor dataset shows the solution implemented in a WiFi-based, high-dimensional scenario. The SmartCampus dataset shows the behavior in a BLE-based scenario. Furthermore, finally, the synthetic dataset simulates a very large-scale scenario, something difficult to implement in a real-world testbed. In the case of both real-world datasets, they were collected using multiple devices (55 different devices in SmartCampus and 25 in UJI Indoor).

In some cases, we had to make some preliminary analyses to find and adjust the best parameters for the HNSW solution. These analyses were all based on the classic ten-fold cross-validation technique [51], which is able to find the best parameters without overfitting the model. In a real-world application, this same validation technique can be used to find the best parameters.

Regarding the implementation and usage of an RSSI simulator based on the indoor signal propagation model, it is important to note that our main goal was to evaluate the scalability of the solution. However, we also take into consideration the resulting error, and thus, it raises the question of how realistic the simulator is. The propagation model used was evaluated in four different buildings in [54], showing its good accuracy when compared to the real world. Furthermore, in [58], the authors use a similar simulator to propose a new IPS that does not require any real-world training, showing that the simulated RSSI values can be used to locate devices in the real world. Thus, we believe that the behavior of the positioning error from our simulator would be at least similar to the ones observed in a real-world application.

## 6. Conclusions

High efficiency is a key factor toward a precise and scalable indoor positioning system. It allows the estimation of locations in real-time by being able to compute thousands or millions of positions every second. Based on some recent work that has shown significant advances in the use of graph-based methods to look for the nearest neighbors in high-dimensional datasets, in this work we proposed the use of Hierarchical Navigable Small World graphs as an optimized data structure to be used in IPSs. We also created a simulation-based synthetic dataset and implemented a complete IPS testbed in a school building to evaluate the performance of the experimented solutions under different conditions.

Our results clearly show the performance gains of the proposed graph-based solution since it was able to estimate the position of the nodes 98% faster than the classic brute force kNN and at least 80% faster than the tree-based optimizations. All of these performance gains were obtained while maintaining almost the same positioning error in all of the tested synthetic and real-world scenario datasets.

Since our solution does not require the loss of information on the training database, some current techniques proposed in the literature to improve accuracy and performance can still be applied in addiition to the solution. However, as mentioned, we did not evaluate the performance of HNSW when combined with other known techniques such as crowdsourcing or computer vision. This would be an interesting aspect to explore in future works.

Furthermore, some other advantages can be further exploited in future works. For instance, we intend to use the knowledge of the last known position of the mobile device, a datum readily available in IPSs, to reach the ground layer of the hierarchical navigable small world graph even faster, allowing for an almost instantaneous location of a node that has been located before.

## Figures and Tables

**Table 1:** Comparison among the three experimented IPS datasets.

Positioning Dataset/Feature	Synthetic	SmartCampus	UJI
Number of Routers	196	42	520
Number of Rooms	378	60	123
Number of Training Points	5390	488	493
Number of Samples per TPs	50	∼2000 (avg)	∼10 (avg)
Total Number of Samples	∼270,000	∼1,020,000	∼21,000

## Data Availability

Not applicable.
